# Analysis of fungal diversity in the gut feces of wild takin (*Budorcas taxicolor*)

**DOI:** 10.3389/fmicb.2024.1364486

**Published:** 2024-04-18

**Authors:** Xiaoping Ma, Zhiguo Li, Lijun Cai, Mei Xiao, Fang He, Zhen Liu, Dong Chen, Ya Wang, Limin Shen, Yu Gu

**Affiliations:** ^1^Key Laboratory of Animal Disease and Human Health of Sichuan Province, College of Veterinary Medicine, Sichuan Agricultural University, Chengdu, China; ^2^Management Office of Tangjiahe National Nature Reserve, Qingchuan, China; ^3^Sichuan Provincial Center for Animal Disease Prevention and Control, Chengdu, China; ^4^College of Life Sciences, Sichuan Agricultural University, Chengdu, China

**Keywords:** *Budorcas taxicolor*, gut fungal diversity, ITS rRNA, amplicon sequencing, metagenomics, opportunistic pathogenic fungi

## Abstract

**Introduction:**

The composition of the intestinal microbiome correlates significantly with an animal’s health status. Hence, this indicator is highly important and sensitive for protecting endangered animals. However, data regarding the fungal diversity of the wild *Budorcas taxicolor* (takin) gut remain scarce. Therefore, this study analyzes the fungal diversity, community structure, and pathogen composition in the feces of wild *B. taxicolor*.

**Methods:**

To ensure comprehensive data analyses, we collected 82 fecal samples from five geographical sites. Amplicon sequencing of the internal transcribed spacer (ITS) rRNA was used to assess fecal core microbiota and potential pathogens to determine whether the microflora composition is related to geographical location or diet. We further validated the ITS rRNA sequencing results via amplicon metagenomic sequencing and culturing of fecal fungi.

**Results and discussion:**

The fungal diversity in the feces of wild *Budorcas taxicolor* primarily comprised three phyla (99.69%): Ascomycota (82.19%), Fungi_unclassified (10.37%), and Basidiomycota (7.13%). At the genus level, the predominant fungi included *Thelebolus* (30.93%), *Functional_unclassified* (15.35%), and *Ascomycota_unclassified* (10.37%). Within these genera, certain strains exhibit pathogenic properties, such as *Thelebolus*, *Cryptococcus*, *Trichosporon*, *Candida*, *Zopfiella*, and *Podospora*. Collectively, this study offers valuable information for evaluating the health status of *B. taxicolor* and formulating protective strategies.

## 1 Introduction

The takin (*Budorcas taxicolor*) is a large hoofed bovid herbivore of the Himalayan region, belonging to the Bovidae family and Caprinae subfamily (Li et al., 2022). The species comprises four subspecies, namely the golden takin (*Budorcas taxicolor bedfordi*), Sichuan takin (*Budorcas taxicolor tibetana*), Bhutan takin (*Budorcas taxicolor whitei*), and Mishmi takin (*Budorcas taxicolor taxicolor*), distinguished according to their morphology and geographical distribution (Chen et al., 2017). The Sichuan takin and Golden takin are unique to China (Zhu et al., 2018); meanwhile, *B. taxicolor* has been listed as an endangered and vulnerable species by the International Union for Conservation of Nature (IUCN) (Ju et al., 2021). Like many wild animals, wild *B. taxicolor* is under pressure, particularly due to hunting and habitat loss caused by rapid population growth (Santymire et al., 2021).

The digestive tract of animals harbors myriad microorganisms (Xue et al., 2015). Indeed, the gastrointestinal tract (GIT) is one of the most complex and rapidly evolving biotic networks responsible for animal functional health (Al Jassim and Andrews, 2009). Fungi contribute to dietary changes by producing cellulolytic and hemicellulolytic enzymes that enhance the capacity for digesting leaves. However, the feces microbiome also affects immune responses by inhibiting or promoting local inflammatory responses (Iliev et al., 2012). Although intestinal fungi are closely associated with animal health, the composition and diversity of intestinal fungi remain unknown for many herbivores.

According to anatomical records from the Chengdu Zoo, the *B. taxicolor* is similar in size to the wild yak (*Bos grunniens*) and gaur (*Bos gaurus*), while its digestive system has characteristics similar to those of sheep. The Sichuan takin is a social ruminant, feeding primarily on trees and shrubs (Kang et al., 2018). As a large ruminant, *B. taxicolor* has a unique digestive system comprising four gastric cavities and relatively long lower feces, suitable for feed rich in plant cell walls (Zhu et al., 2018). The bulk of current literature addresses only ruminant fermentation (Mura et al., 2019). Although the bacterial community composition of *B. taxicolor* has received considerable research attention, the characterization of fungal colonization has been relatively neglected (Guan et al., 2023; Ma et al., 2023).

Although the microbiome composition and functions remain stable over extended periods, a multitude of factors significantly impact these properties. These include but are not limited to dietary factors, geographical location, age, genetics, mode of delivery, and medical treatments (Panda et al., 2014). Accordingly, we hypothesize that the composition of intestinal fungi may be related to geographical location or diet. To test this hypothesis, we analyzed the composition and potential pathogens in the intestinal feces of *B. taxicolor*. The findings of this study provide new insights into the composition of intestinal fungi while supplementing existing knowledge on their assumed role in health. The finding will help protect this species by providing vital information.

## 2 Materials and methods

### 2.1 Sampling

Samples were collected from the Sichuan Tangjiahe National Nature Reserve, China (32°59′N, 104°77′E). A total of 82 fecal samples were collected from areas A (104°809′–104°849′E, 32°5444′–32°589′N), B (104°821′–104°867′E, 32°624′–32°649′N), C (104°761′–104°807′E, 32°542′–32°591′N), D (104°643′–104°686′E, 32°578′–32°611′N), and E (104°690′–104°743′E, 32°578′–32°613′N), designated GI, GII, GIII, GIV, and GV, respectively. These sites have similar climates, with differences in vegetation caused by variations in elevation. During collection, feces were collected in sterile tubes to prevent direct contact with the surface; new tools were used for each sample (Yu C. et al., 2021). All samples were kept in a portable incubator filled with dry ice during the field investigation. Samples were stored in an insulated container with dry ice and transported to the laboratory within 2 h of collection for isolation and culture (Ma et al., 2024).

### 2.2 DNA extraction

Cetyltrimethyl ammonium bromide (CTAB) effectively extracts fungal DNA from microscopic samples (Bansal et al., 2018). Thus, CTAB (Solarbio, Beijing, China) was used to extract DNA from different samples according to the manufacturer’s instructions. Nuclease-free water was used for the negative controls. Total DNA was eluted in 50 μL of elution buffer (Sangon Biotech, Shanghai, China) and stored at −80°C for PCR analysis, which was performed by LC-Bio Technology Co., Ltd., Hang Zhou, Zhejiang Province, China. The associated results were applied for subsequent amplicon sequencing of internal transcribed spacer (ITS) rRNA and amplicon metagenomic sequencing.

### 2.3 PCR amplification and ITS sequencing for data analysis

Each sample underwent labeling of the 5′ terminus of the primer with a unique barcode and sequencing with a common primer (Kumari et al., 2016). PCR amplification was performed containing 12 μL of PCR premix, 25 ng of template DNA, 2 μL of each primer, and PCR-grade water to adjust the final reaction volume to 25 μL (Roggenbuck et al., 2014). The PCR amplification conditions comprised an initial denaturation at 98°C for 30 s, followed by 32 cycles of denaturation at 98°C for 10 s, annealing at 54°C for 30 s, and extension at 72°C for 45 s. The final extension step was conducted at 72°C for 10 min (Li, 2023). The PCR results were verified using agarose gel electrophoresis on a 2% agarose gel. Ultrapure water was used throughout the PCR process to prevent false positives. The PCR products were purified using AMPure XT beads (Beckman Coulter Genomics, Danvers, MA, United States). Quantification was performed with Qubit (Invitrogen, United States). The size and quantity of the amplification pool were assessed using Agilent 2100 Bioanalyzer (Agilent, United States) and the Library Quantification Kit for Illumina (Kapa Biosciences, Woburn, MA, United States), respectively. Subsequently, the libraries were sequenced on the NovaSeq PE250 platform (Agwunobi et al., 2022) to obtain the desired results.

### 2.4 Data analysis

Per the manufacturer’s guidelines, the Illumina NovaSeq platform provided by LC-Bio was used to sequence the samples. Each sample was assigned to individual paired-end reads based on its unique barcode. This was followed by trimming of the barcodes and primer sequences. High-quality reads were obtained using fqtrim (v0.94) with specific filtering criteria. Vsearch software (v2.3.4) was employed to identify and eliminate chimeric sequences. Using DADA2, we generated a feature table and sequences (Caporaso et al., 2010). The alpha and beta diversity calculations were performed using QIIME2 (Poncheewin et al., 2019). The samples’ complexity and alpha diversity were assessed based on five indices: Goods_coverage, Chao1, observed species, Simpson, and Shannon. This involved minimizing the sequence count in selected samples, randomly extracting an equal number of sequences, and using relative abundance for fungal classification (X fungal count/total count). The figures were generated using R (v3.5.2) (Ritchie et al., 2015). To annotate the species, the QIIME2 feature classifier was applied to compare sequences against the Ribosomal Database Project (RDP) database^1^ (Maidak et al., 1997). The unigene functional annotations (KEGG) were obtained. Taxonomic, functional, and gene-wise differential analysis was performed using Fisher’s precise test (non-replicated groups) or the Kruskal–Wallis test (replicated groups) based on the taxonomic and functional annotations and abundance profile of the unigenes.

### 2.5 Isolation and identification of fungus

Feces were inoculated on Sabouraud medium (SDA) with chloramphenicol and Actidione using a disposable aseptic inoculation ring. The plates were incubated at 25°C, and fungal growth was observed every 12 h. We selected different fungal colonies based on their unique colonial morphologies until a single colony was obtained through purification. Fungal DNA was extracted using the Rapid Fungi Genomic DNA Isolation Kit (Sangon Biotech, Shanghai, China). PCR amplification of the 18S region was conducted using universal primers ITS 1 (5′-TCCGTAGGTGAACCTGCGG-3′) and ITS4 (5′-TCCTCCGCTTATT-GATATGC-3′) (Ahmad et al., 2022). The PCR mixture contained 1.0 μL of DNA template, 12.0 μL of Premix Taq (Ex TaqVersion 2.0 plus dye), 1.0 μL of forward and reverse primers each, and 10 μL of ddH_2_O. The PCR procedure involved 94°C for 10 min, 94°C for 30 s, 55°C for 30 s, 72°C for 1 min 10 s, 30 cycles, and 72°C for 5 min. The PCR products were Sanger sequenced by Sangon Biotech (Shanghai, China), and the sequencing data were subjected to NCBI BLAST analysis (Ma et al., 2024). The obtained data were compared with the sequence data in the NCBI GenBank. MEGA5 software was used to construct contrasting sequencing trees (Ma et al., 2023).

## 3 Results

### 3.1 Sequencing data

The sequencing non-zero value was 0.033; a total of 6,799,161 reads were sequenced from 82 samples, with 6,515,078 effective sequences retained after filtering by double-ended splicing, quality control, and chimera removal. Each group of samples produced a minimum of 1,136,430 raw reads, representing an average of 80,433 effective sequences. The reads were predominantly 200–400 bp in length. A total of 13,280 amplicon sequence variants (AVSs) were detected.

### 3.2 Venn map analysis

**Figure 1:**
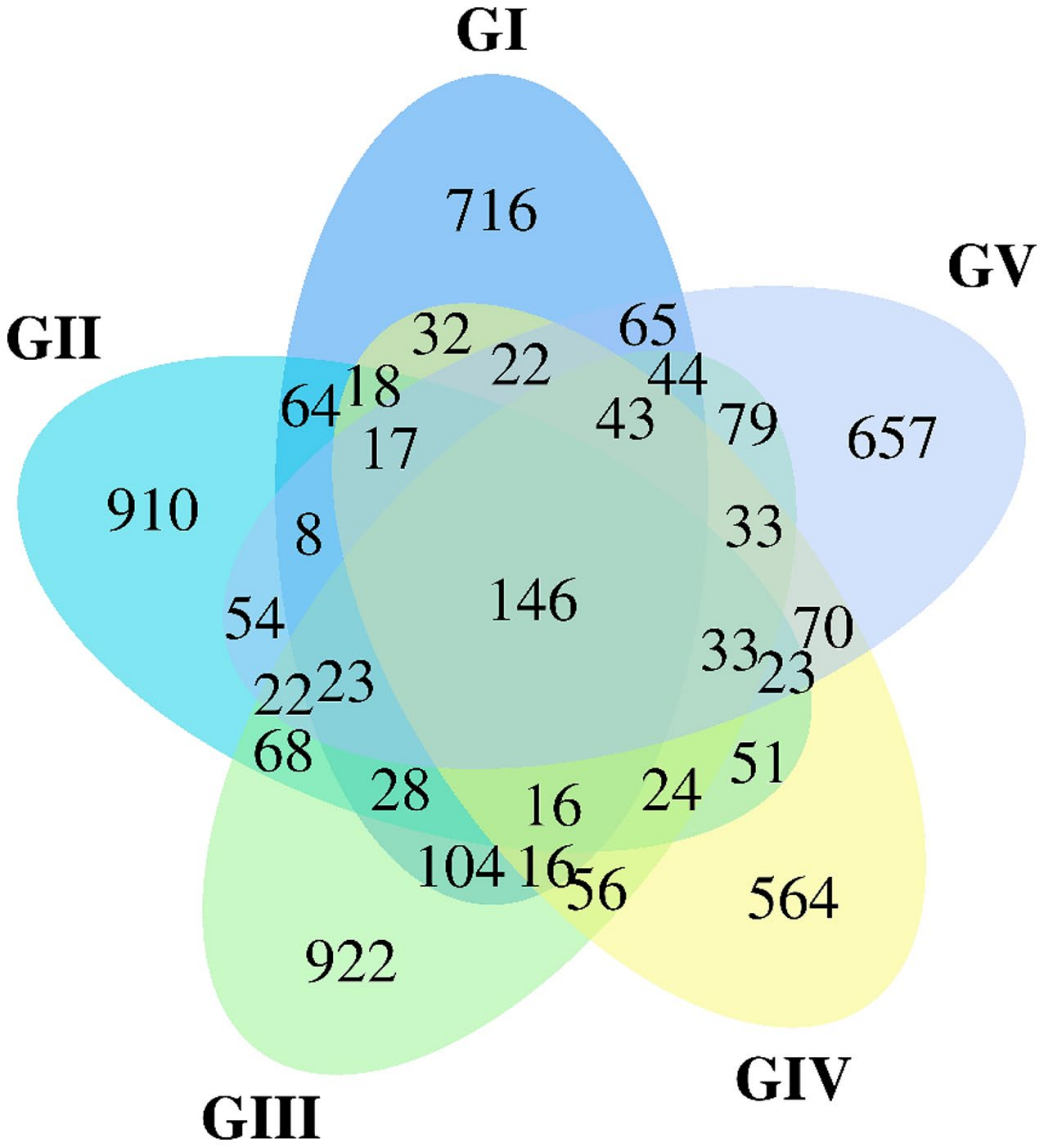
Venn diagram of group GI vs. GII vs. GIII vs. GIV vs. GV. A Venn diagram is used to visualize the number of ASVs that are common and specific to each sample.

The Venn map was created based on the number of AVSs detected in *B. taxicolor* feces. Cluster analysis showed 1,362, 1,505, 1,657, 1,164, and 1,339 AVSs in GI, GII, GIII, GIV, and GV samples, accounting for 10.26, 11.33, 12.48, 8.77 and 10.08%, of the total AVSs, respectively. The number of unique AVSs in each of the five groups was 716, 910, 922, 564, and 657, respectively, accounting for 5.39, 6.85, 6.94, 4.25, and 4.95% of the total AVSs, respectively. Meanwhile, 146 AVSs common to all five groups were obtained, accounting for 1.10% of the total (Figure 1).

### 3.3 Alpha diversity comparison of the five groups

**Figure 2:**
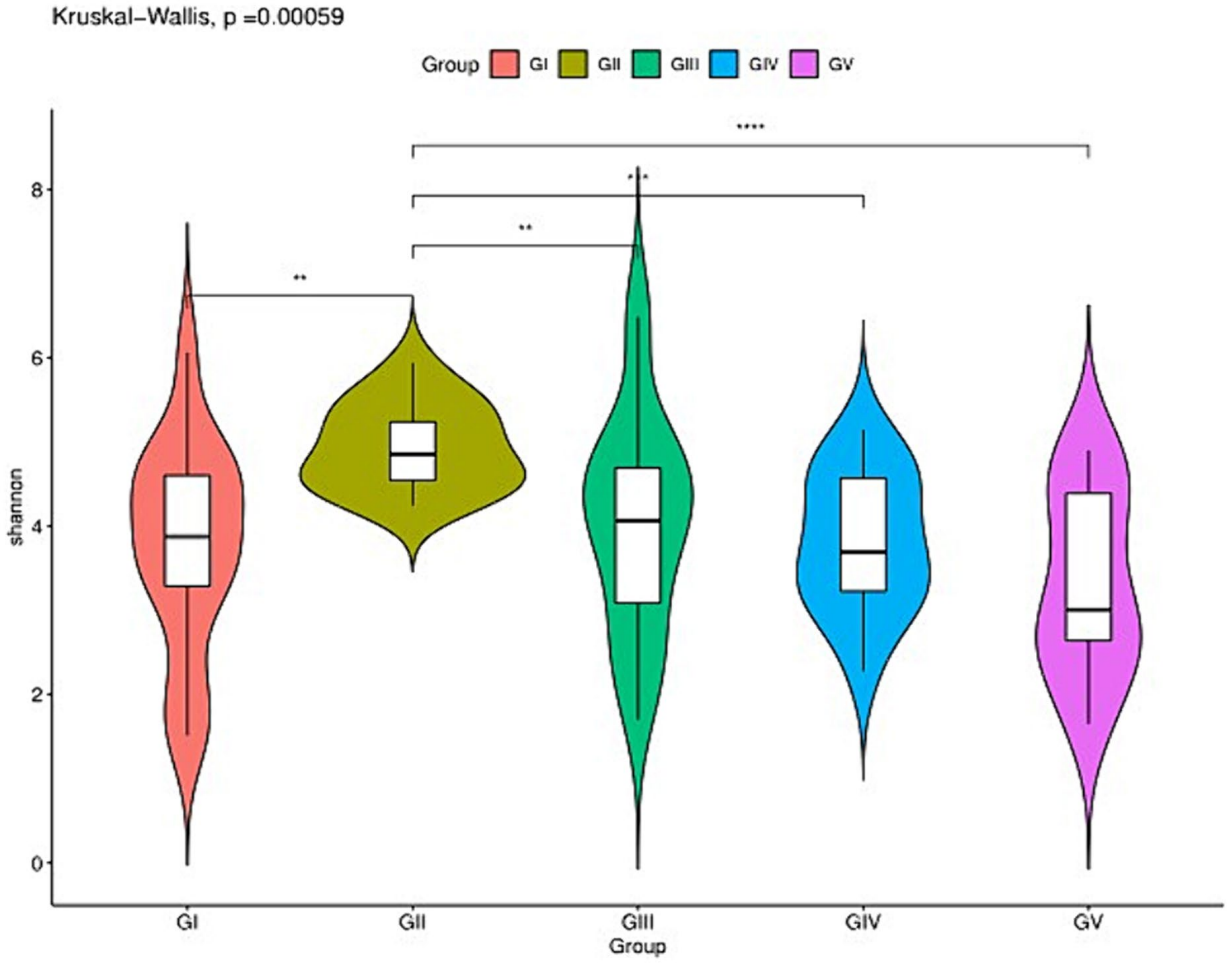
Species richness of feces fungi of the wild *Budorcas taxicolor* measured via ITS rDNA sequencing (^*^p < 0.05, ^**^p < 0.01, ^***^p < 0.001, and ^****^p < 0.0001). Comparison of Shannon index between groups GI, GII, GIII, GIV, and GV. ^*^Means significant difference, ^**^indicates extremely significant difference and ns indicates no significant difference.

The analysis of the alpha diversity between GI, GII, GIII, GIV, and GV is shown in Supplementary Table S2 and Figure 2. The fungal diversity decreased in the order of GII > GIII > GIV > GI > GV based on the Shannon index (4.88 vs. 4.01 vs. 3.81 vs. 3.70 vs. 3.33) (Supplementary Table S1). The evenness of fungi decreased in the order of GII > GIII > GIV > GI > GV, based on the Pielou index (0.65 vs. 0.55 vs. 0.53 vs. 0.50 vs. 0.46). The rank abundance curve revealed a rich and uniform representation (Supplementary Figure S1). The fungal diversity and evenness decreased in the order of GII > GIII > GIV > GI > GV. GII had the highest fungal diversity, while GV had the lowest (Figure 2).

### 3.4 Community-composition analysis

**Figure 3:**
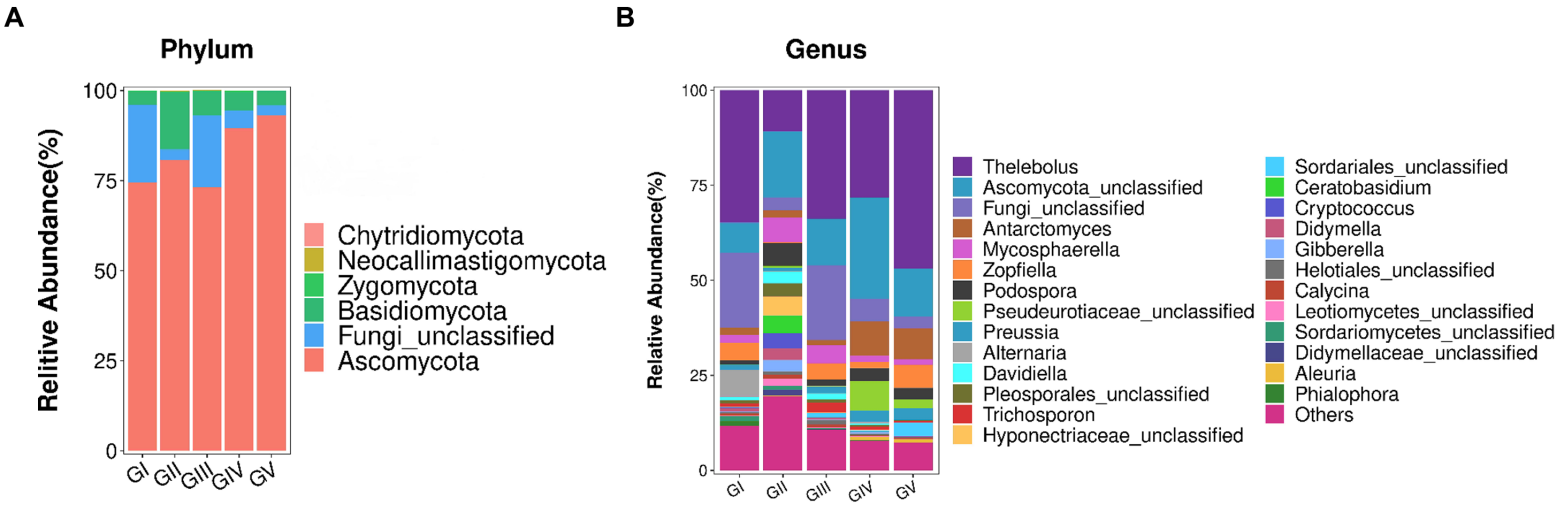
**(A)** Analysis of fungal community structure at the phylum level. The abscissa is the phylum classification, and the ordinate is the relative abundance percentage. **(B)** Analysis of the fungal community structure at the genus level. At the genus level, all genera with effective sequence abundances within the top 25 were screened for statistical analysis. The abscissa is the genus classification, and the ordinate is the relative abundance percentage.

The fungal AVSs were classified into 6 phyla, 27 classes, 88 orders, 198 families, 431 genera, and 755 species. While the kingdom, phylum, class, order, family, genus, and species levels were included in the study, data was only analyzed at the phylum and genus levels (Figure 3). At the phylum level, the valid sequences of the five groups of samples were assigned to six phyla: Ascomycota (82.19%), Fungi_unclassified (10.37%), Basidiomycota (7.13%), Zygomycota (0.17%), Neocallimastigomycota (0.12%), and Chytridiomycota (0.01%). Ascomycota was the most abundant phylum, followed by Fungi_Unclassified and Basidiomycota. According to the amplicon metagenomic sequencing results, Eukaryota_unclassified, Ascomycota, and Basidiomycota were the dominant phyla. This was consistent with the amplicon sequencing of ITS rRNA results (Supplementary Figure S2). In the metagenomic grouping, salt-licking *B. taxicolor* were assigned to the GN group (saline region), while the normal *B. taxicolor* were assigned to the GW group (unsalted region).

The top 25 fungal genera, based on the number of assigned reads, were screened from the fungal communities (Supplementary Table S2). Using cluster analysis, we distinguished the abundances of different species. In the five groups of samples, *Thelebolus* comprised the largest proportion (30.93%), followed by *Functional_unclassified* (15.35%) and *Ascomycota_unclassified* (10.37%).

The amplicon metagenomic sequencing confirmed the above results. Supplementary Figure S3 shows a certain proportion of *Anaeromyces* that was also shown in Figure 3B, while *Aspergillus* was also isolated. *Thelebolus*, *Functional_unclassified,* and *Ascomycota_unclassified* may belong to the *Eukaryota_unclassified* genera in amplicon metagenomic sequencing.

### 3.5 Fungi isolation and identification

A total of 10 fungal species were identified, including *Fragosphaeria purpurea*, *Cladosporium cladosporioides*, *Purpureocillium lilacinum, Penicillium aeneum*, *Penicillium citreonigrum*, *Aspergillus*, *Trichosporon asahii*, *Candida albicans*, *Trichosporon lactis*, and *Beaureria bassiana*. The Community Composition Analysis indicated that *Fungi_unclassified* comprised a significant proportion of the species. This likely included the aforementioned fungi. Moreover, the identification of *Trichosporon* confirmed the Community Composition Analysis findings. The evolutionary tree of the fungi is shown in Supplementary Figure S4. The NCBI accession numbers are presented in Supplementary Table S3.

### 3.6 Characterization of microbial taxa associated with GI, GII, GIII, GIV, and GV

**Figure 4:**
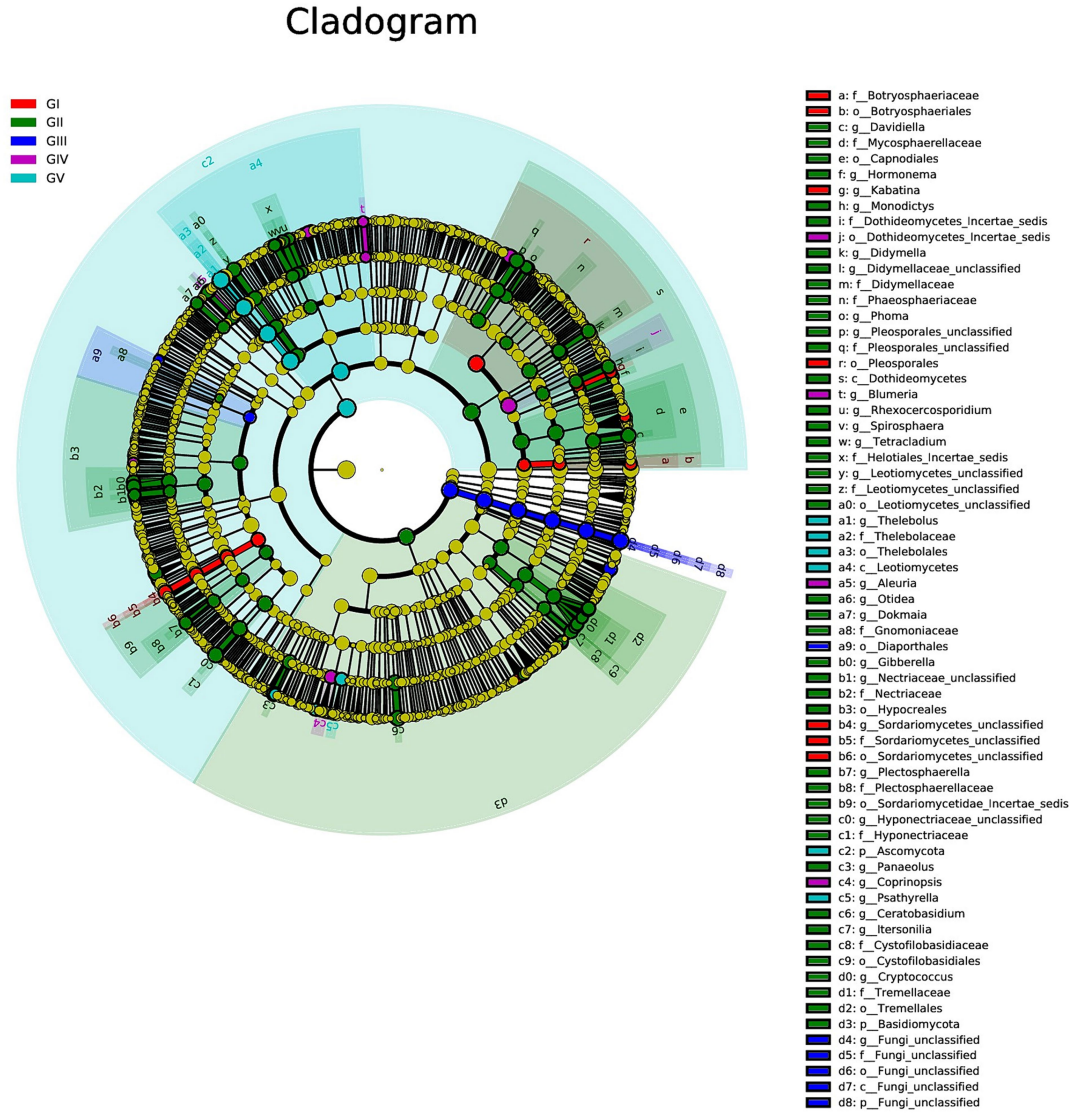
Results of linear discriminant analysis effect size (LEfSe) analysis revealing the most abundant taxa from the phylum to the genus level among GI, GII, GIII, GIV, and GV. The red, green, blue, purple, and turquoise areas represent groups GI, GII, GIII, GIV, and GV, respectively. In the cladogram, the red, green, blue, purple, and turquoise nodes represent the main roles of the groups A, B, and C. In each LDA, strains that do not function primarily have corresponding yellow nodules, which indicated that LDA can reflect the influence degree of different species in different populations. Species that differ significantly with LDA scores greater than the preset value are shown; statistically significant biomarkers are shown with a default preset value of 3.0.

We used multilevel LEfSe analysis to identify possible and meaningful microbial biomarkers from various groups. Considering the diversity of fungi, those with LDA scores >3.0 were selected as the main research objects (Figure 4).

At the genus level, *Kabatina* and *Sordariomycetes_unclassified* were identified as strong markers in GI. *Davidiella*, *Hormonema*, *Monodictys*, Dothideomycetes, incertae sedis, *Didymella*, and others were identified as markers in GII, whereas fungi_unclassified was identified as a marker in GIII. Meanwhile, *Blumeria* and *Aleuria* were identified as markers in GIV, and *Thelebolus* was identified as a marker in GV (Figure 4; Supplementary Figure S5). It was further confirmed that GII had the highest fungal diversity.

**Figure 5:**
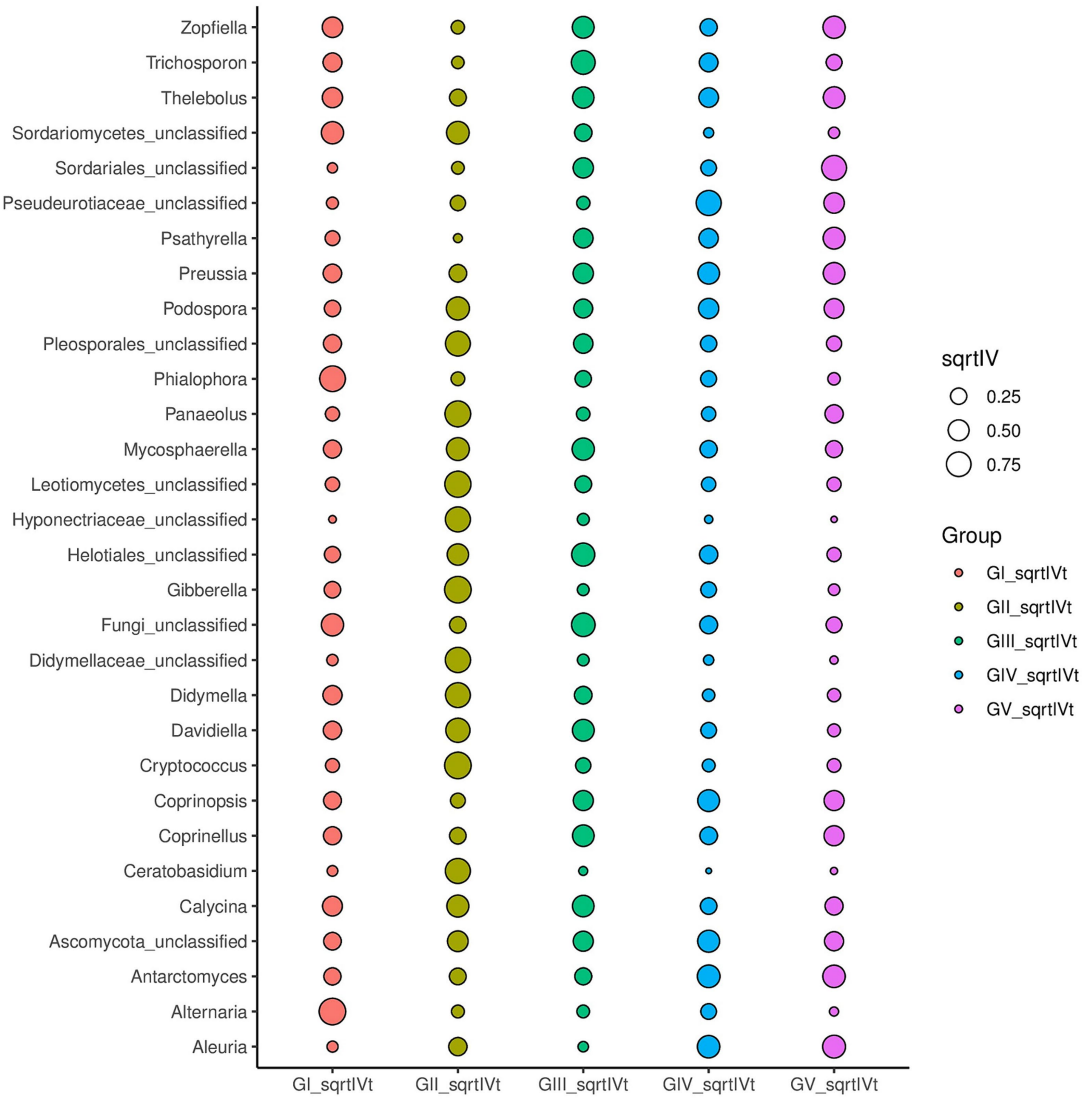
For different taxonomic levels, the top 30 abundant species are applied to identify different groups of species to be used as biomarkers; sqrtIVt value is the square root of the indicator value, used to compare the correlation between microorganisms and treatment groups. The greater the value, the better the species will perform as biomarkers of the treatment group.

Indicator analysis revealed *Alternaria* and *Phialophora* in GI, *Cryptococcus* and *Davidiella* in GII, *Thelebolus*, *Davidiella*, and *Trichosporon* in GIII, and *Thelebolus* in GV as fungi with sqrtIVt values >0.5. Hence, these fungi may serve as potential indicator species for disease detection (Figure 5). Additionally, various soil saprophytic fungi and plant pathogens were identified.

### 3.7 Functional analysis of GI, GII, GIII, GIV, and GV

**Figure 6:**
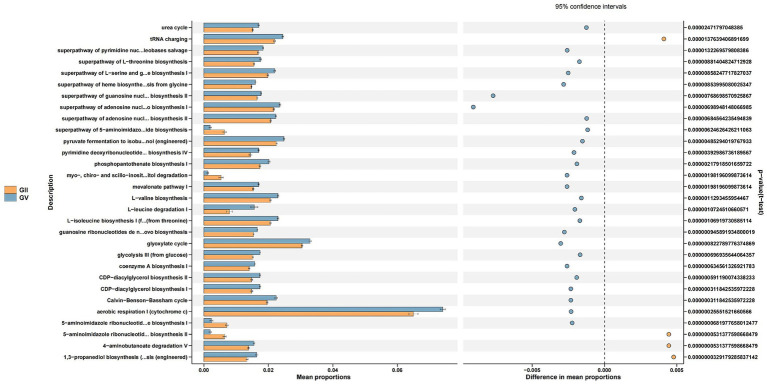
Differential Kyoto Encyclopedia of Genes and Genomes (KEGG) pathways analyzed using PICRUSt for the GII and GV.

Through the Kyoto Encyclopedia of Genes and Genomes (KEGG) pathway analysis, the effects of intestinal microflora on body function were determined (Douglas et al., 2020). Among the common pathways of the five groups, significant differences were primarily detected in replication repair, translation, and nucleotide metabolism and were predominantly observed in group GII. Specifically, compared with GI, GII, and GIII, genes associated with metabolism were identified in GII samples, which may relate to disease (Figure 6; Supplementary Figures S6–S8).

## 4 Discussion

*Budorcas taxicolor* is a class I protected animal in China that is listed as an endangered species in the Red Book. To date, China has taken various protective measures, including establishing nature reserves and implementing captive breeding. However, these strategies have ignored the effect of intestinal fungi in wild Takin.

In the current study, data was analyzed based on the RDP database, which provides 28S rRNA sequences for Fungi (Eukarya) (Balvočiūtė and Huson, 2017). RDP has +97% assignment accuracy and provides rapid results for reads that are 250 bp or longer when they originate from a taxon known to the database (Lan et al., 2012). Indeed, the RDP classifier has been employed to correlate mammals to their gut microbes (Ley et al., 2008). However, in the current study, most annotations were limited to genera due to the short reads (<250 bp), impeding the investigation of detailed species. Moreover, fungi have received relatively less research attention. In our study, the fraction of non-zero values was only 0.033. Additionally, given that fungi account for ~0.1% of the gut (Qin et al., 2010; Arumugam et al., 2011), many fungal classifications have not yet been annotated in the RDP database. This limitation hinders research, particularly in non-human species. Accordingly, the amplicon sequencing of ITS rRNA results were verified by amplicon metagenomic sequencing and isolation cultures. *Aspergillus* and *Trichosporon* from isolation culture and amplicon metagenomic sequencing validated the ITS rRNA sequencing results.

Alpha diversity analysis was employed to evaluate and compare the species diversity of individual plots. As the curve becomes flat, the quantity of sequencing data tends to be reasonable, and the homogeneity among samples is high (Kleine Bardenhorst et al., 2022), indicating that the sample size is adequate for data analysis (Ma et al., 2021). The differences in the Simpson and Shannon indices between the five groups were significant (*p* < 0.05). GII had the highest abundance, indicating the highest species richness and diversity among the five groups. The AVS count of GII was the highest (Figure 1), which could be attributed to the location and diet. Similar dietary effects were also demonstrated by Zhu et al. (2018) in their analysis of fecal microbial diversity in captive and wild *B. taxicolor* antelope. Moreover, the fungal diversity within the feces of wild *B. taxicolor* was highest in Ascomycota (82.19%) at the phylum level. Similarly, Ascomycota contributed the highest proportion of core feces microbial communities in sheep (Tanca et al., 2017), giraffes (Schmidt et al., 2018), *Cervus nippon* (Hu et al., 2020), and Tibetan macaques (Sun et al., 2018). This phenomenon may arise from Ascomycetes being primarily plant parasites. Additionally, Ascomycota is influenced by fiber content (Pitta et al., 2010). The environment at different sites likely differ regarding food, water, and soil composition. Additionally, certain conservation stations have salt ponds, which may have contributed to the observed differences by increasing the soil salinity (Supplementary Figures S3, S4) (Li et al., 2016).

Twenty-five fungal genera were identified, some of which were potentially pathogenic. When animals age, parasitic infections, traumatic injuries, and other diseases cause their immune function to decrease, making them susceptible to infection by potentially pathogenic fungi. All five groups of samples contained *Thelebolus*, which was the genera; similar findings were reported in yak (Zhu et al., 2023) and beef cattle (Tong et al., 2023) under different feeding patterns. *Thelebolus* negatively impacts ruminant health and behavior (Koester et al., 2020). Mechanistically, *Thelebolus* spp. may influence exopolysaccharide to further induce apoptosis in healthy intestinal cells, thus restricting nutrient uptake and decreasing intestinal absorption.

In addition, Cryptococcus and Trichosporon were detected in our study samples. Cryptococcus (de Freitas et al., 2020) and Trichosporon (Centeno-Martinez et al., 2023) have also been detected in beef cattle. Cryptococcal meningitis may occur in animals infected with cryptococcus (Yu D. et al., 2021), whereas multiple Trichosporons, including *Trichosporon asahii* (Ramírez and Moncada, 2020), can cause fatal Trichospore bacteremia (Basu et al., 2015). Furthermore, *Zopfiella* and *Podospora* were also detected in our study samples. *Zopfiella* (Fujimoto et al., 2004) and *Podospora* (Ying et al., 2021) cause immune suppression. However, the isolation of potentially pathogenic fungi from feces does not provide definitive evidence of a host’s health status (Ma et al., 2022). Moreover, although the distribution of these pathogens in the wild *B. taxicolor* is unknown, they might represent a source of zoonotic infections. However, due to the relative dearth of relevant data, the roles of the fungal community and individual fungal taxa in intestinal physiology remain poorly understood (Hu et al., 2020). In the last 20 years, considerable progress has been made in the study of mammalian intestinal tissue; however, research on the function of microorganisms in the lower feces of ruminants remains at a relatively early stage (O’Hara et al., 2020).

When an animal’s immune system is compromised, opportunistic fungi can become pathogenic. In some cases, the risk of infection increases, especially in a stressed state during which external factors exert adverse effects on the body’s immune system. However, the microbial ecology of feces does not precisely reflect the health of the host as certain animal pathogens are not responsible for causing *B. taxicolor* disease. On the one hand, no targeted etiological studies have been carried out to identify and verify the pathogenesis of these potential pathogens. On the other hand, capturing this animal for further investigation and confirmation has proven impossible for our research team. Nevertheless, this point of view can be put forward for the scientific protection of *B. taxicolor* (Ma et al., 2022).

The comprehensive analysis of KEGG metabolic pathways indicated that most microorganisms were predominantly involved in processes related to replication repair, translation, and nucleotide metabolism. However, others were associated with carbohydrate metabolism, fat metabolism, and energy metabolism. These metabolic processes are an important factor in determining fungal pathogenicity and virulence, due to their essential roles in facilitating the onset and advancement of infectious processes and in governing the generation and functionality of virulence factors, such as the fungal cell wall structure and melanin (Ruijter and Visser, 1997; ter Schure et al., 2000; Ries et al., 2018). The metabolic processes in these fungi significantly contribute to their adaptability, survival, and virulence within their host environment (Ries et al., 2018).

## 5 Conclusion

In this study, we used amplicon sequencing of ITS rRNA to comprehensively analyze the fungal diversity in the feces of wild *B. taxicolor* from five conservation stations. The fungal diversity primarily comprised Ascomycot, Fungi_unclassified, and Basidiomycota at the phyla level. Most microorganisms were found to have critical roles in replication, repair, translation, and nucleotide metabolism. The GII site had the highest species richness and diversity among the five groups, which may be due to differences in location and diet. We also detected potentially pathogenic genera, including *Thelebolus*, *Cryptococcus*, *Trichosporon*, *Zopfiella*, and *Podospora*, providing forewarning to inform the conservation of these endangered species. Although the fecal samples were relatively large, capturing wild *B. taxicolor* was not possible, impeding the evaluation of clinical symptoms and, thus, preventing an in-depth analysis of host health. Future studies should focus on evaluating a larger sample of *B. taxicolor* in terms of study duration and area to achieve a more comprehensive comparative analysis. Nevertheless, our study is the first step toward elucidating the fungal diversity in the feces of wild *B. taxicolor.*

## Data availability statement

The datasets presented in this study can be found in online repositories. The names of the repository/repositories and accession number(s) can be found in the article/Supplementary material.

## Ethics statement

The animal study was approved by Sichuan Agricultural University Animal Ethics Committee and Sichuan Tangjiahe National Nature Reserve (permit number DYYS20211118). The study was conducted in accordance with the local legislation and institutional requirements.

## Author contributions

XM: Formal analysis, Funding acquisition, Investigation, Methodology, Supervision, Writing – original draft, Writing – review & editing. ZoL: Data curation, Formal analysis, Software, Visualization, Writing – original draft. LC: Funding acquisition, Project administration, Resources, Validation, Writing – original draft. MX: Conceptualization, Formal analysis, Investigation, Resources, Writing – original draft. FH: Formal analysis, Investigation, Methodology, Resources, Writing – original draft. ZnL: Methodology, Validation, Visualization, Writing – original draft. DC: Conceptualization, Investigation, Software, Writing – original draft. YW: Investigation, Methodology, Supervision, Writing – review & editing. LS: Funding acquisition, Project administration, Resources, Writing – review & editing. YG: Conceptualization, Data curation, Formal analysis, Supervision, Validation, Writing – review & editing.

## Footnotes

^1^http://rdpwww.life.uiuc.edu/
