# Supplementary figures and images for: Analysis of fungal diversity in the gut feces of wild takin (Budorcas taxicolor)
Source: Front Microbiol. 2024 Apr 18;15:1364486. doi: 10.3389/fmicb.2024.1364486 (PMC11063333; doi:10.3389/fmicb.2024.1364486)

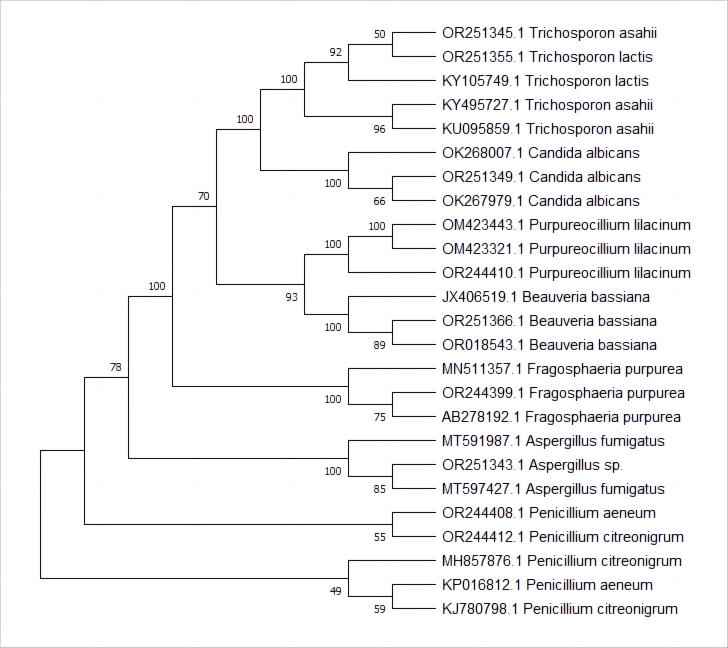

Supplement: Supplementary file 1 [file Image_1.JPEG]

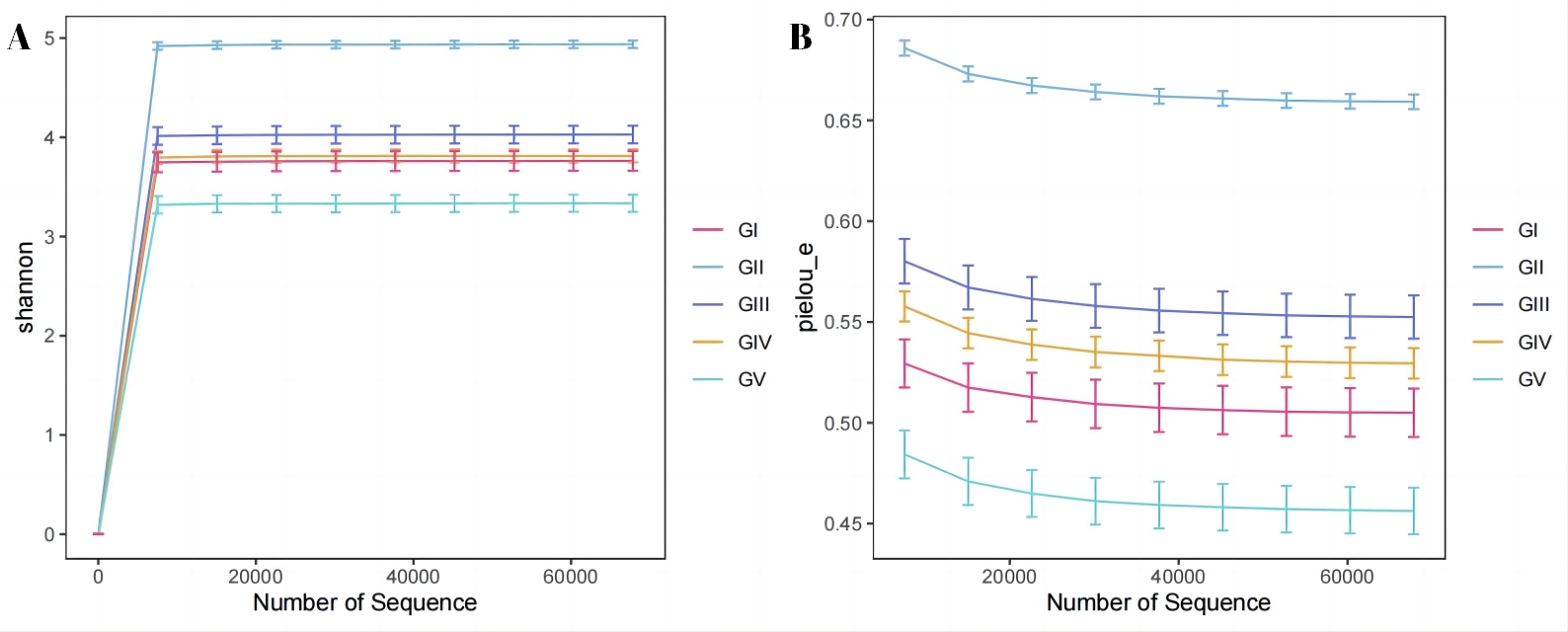

Supplement: Supplementary file 2 [file Image_2.JPEG]

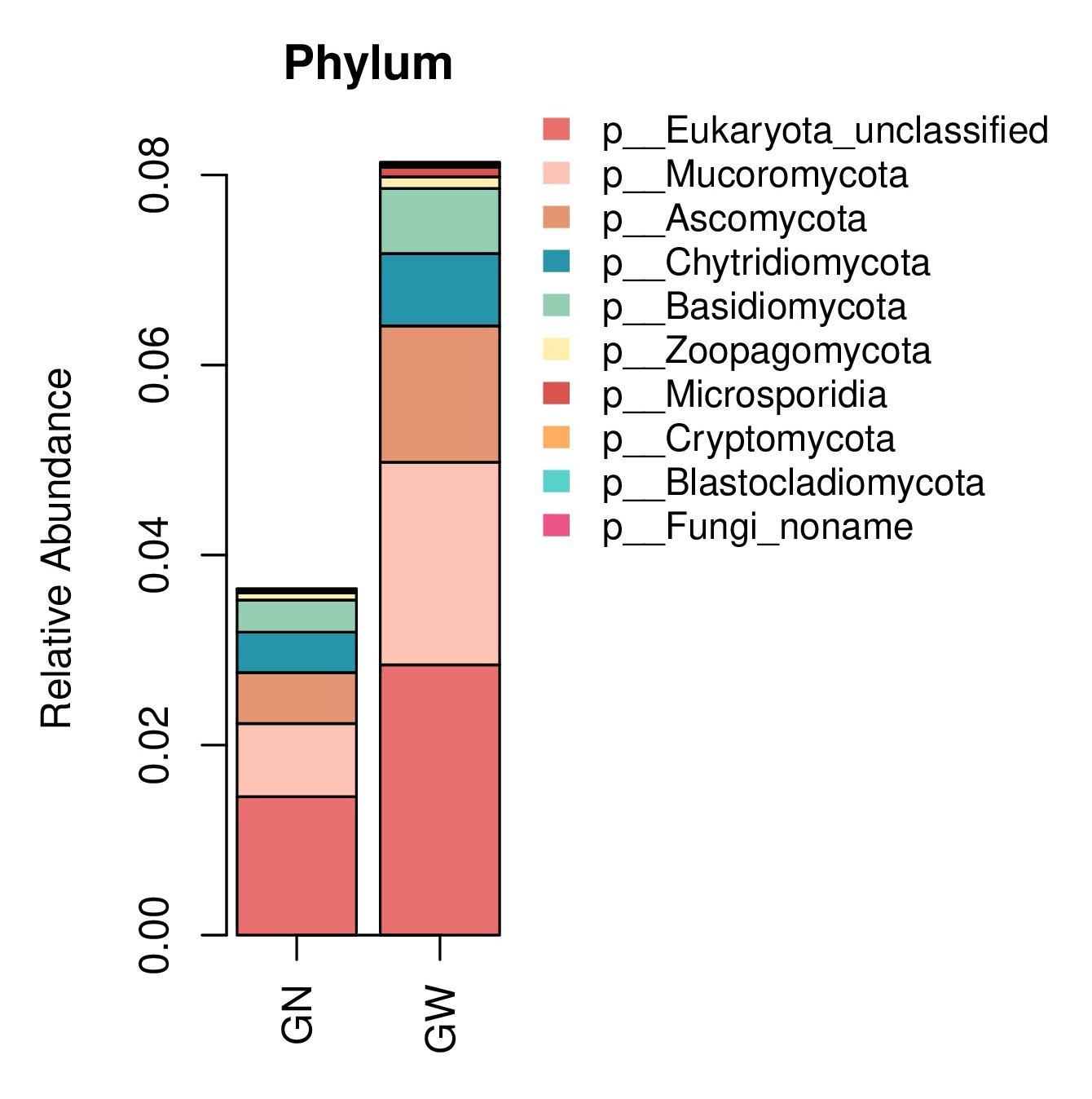

Supplement: Supplementary file 3 [file Image_3.JPEG]

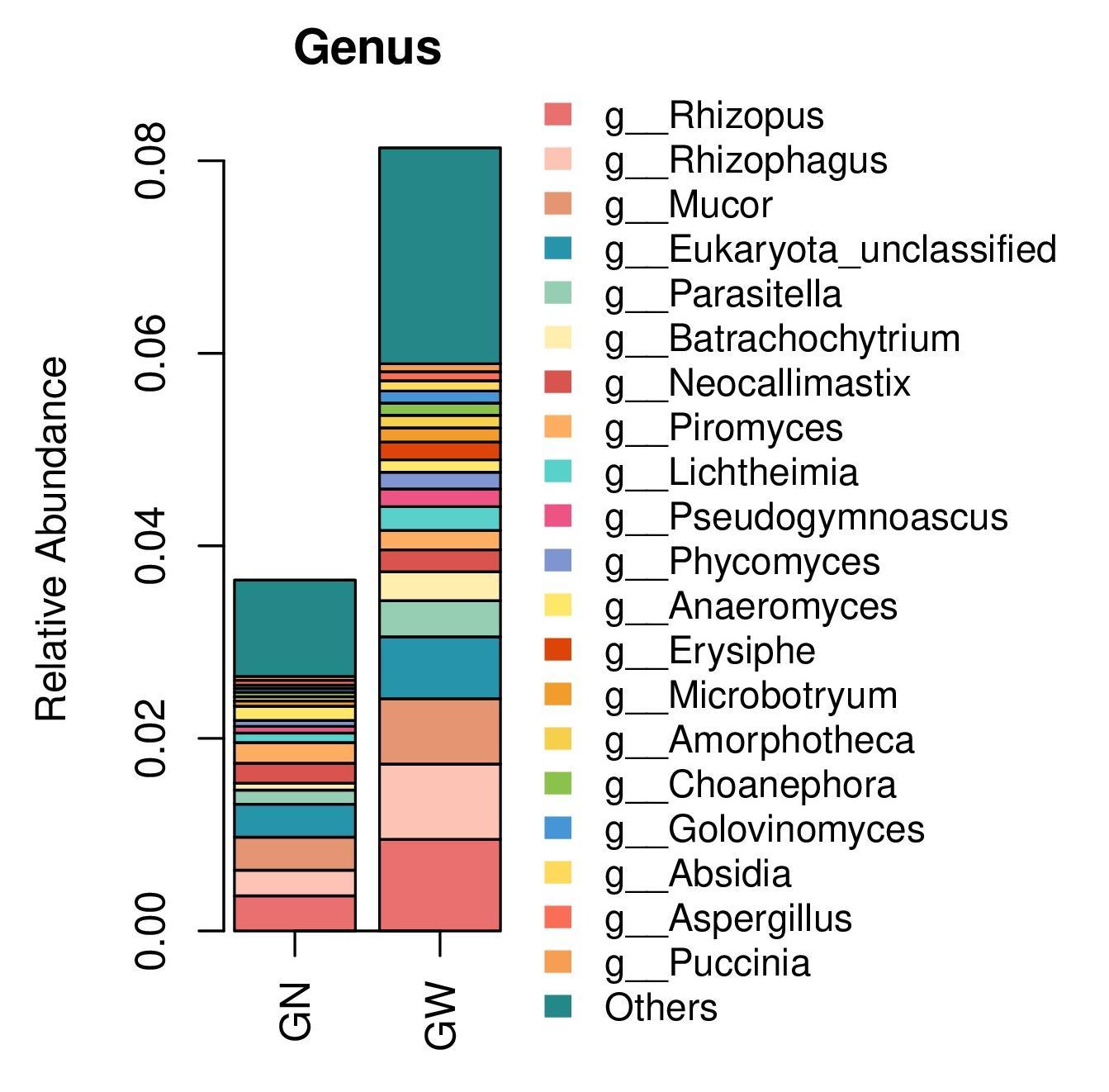

Supplement: Supplementary file 4 [file Image_4.JPEG]

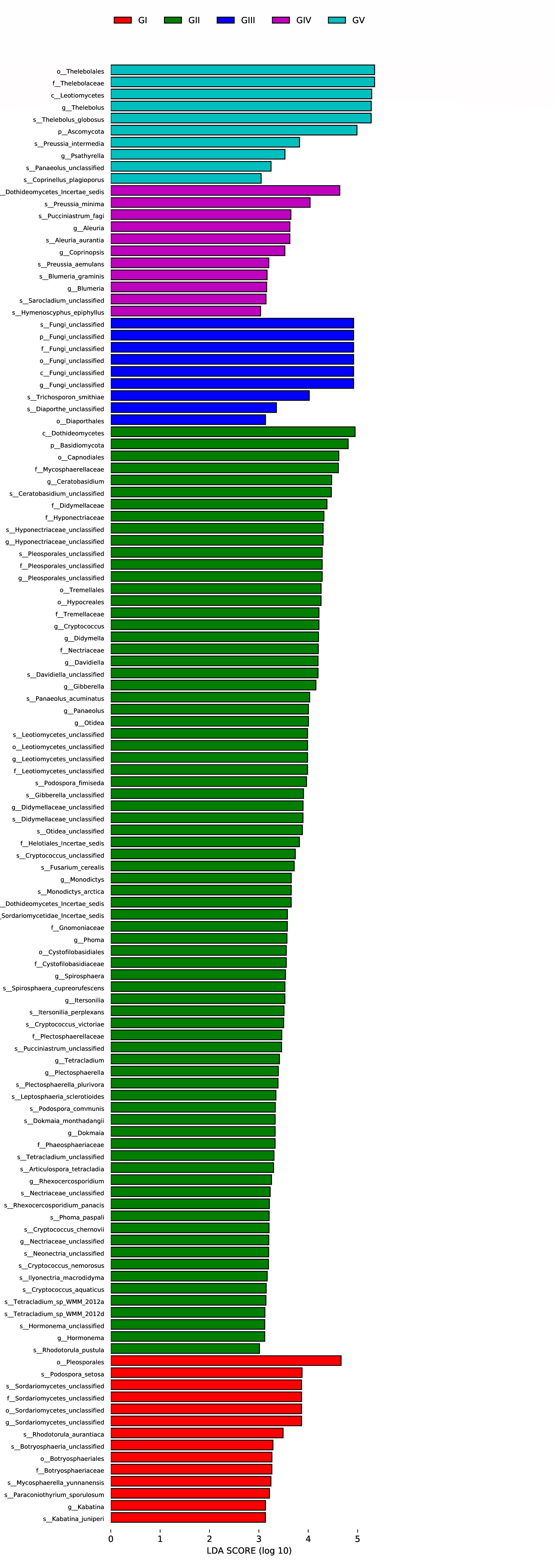

Supplement: Supplementary file 5 [file Image_5.JPEG]

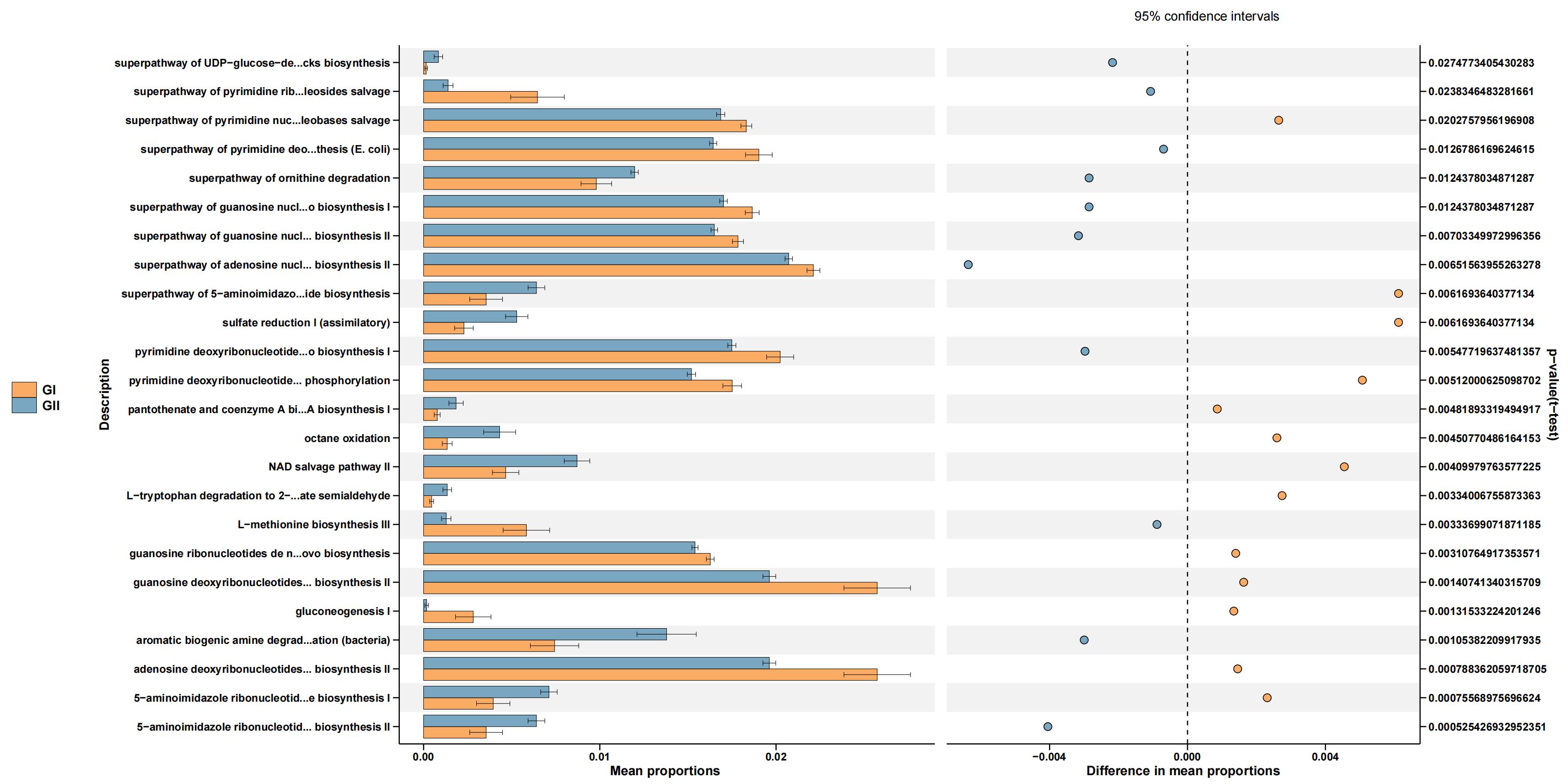

Supplement: Supplementary file 6 [file Image_6.JPEG]

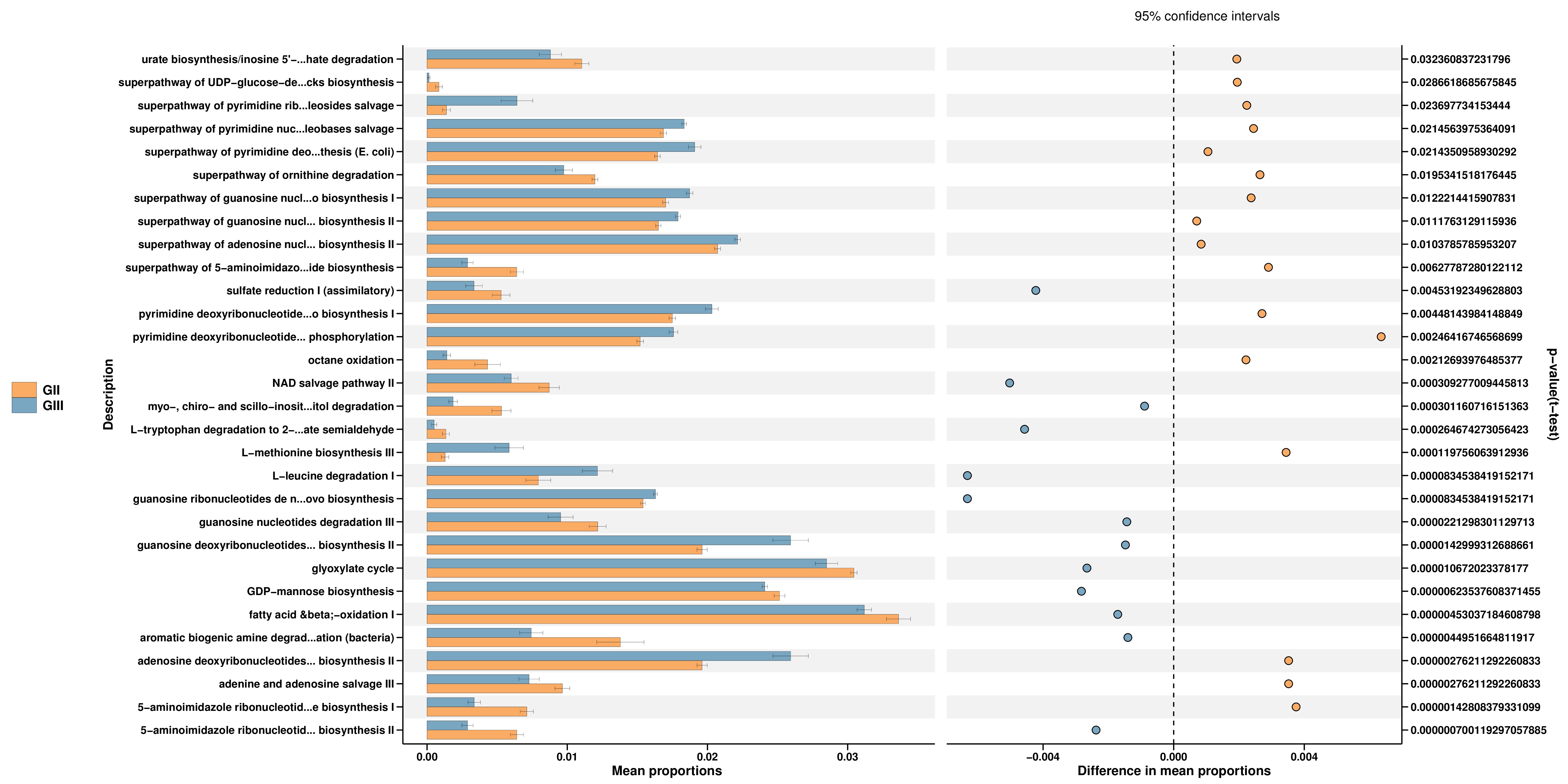

Supplement: Supplementary file 7 [file Image_7.JPEG]

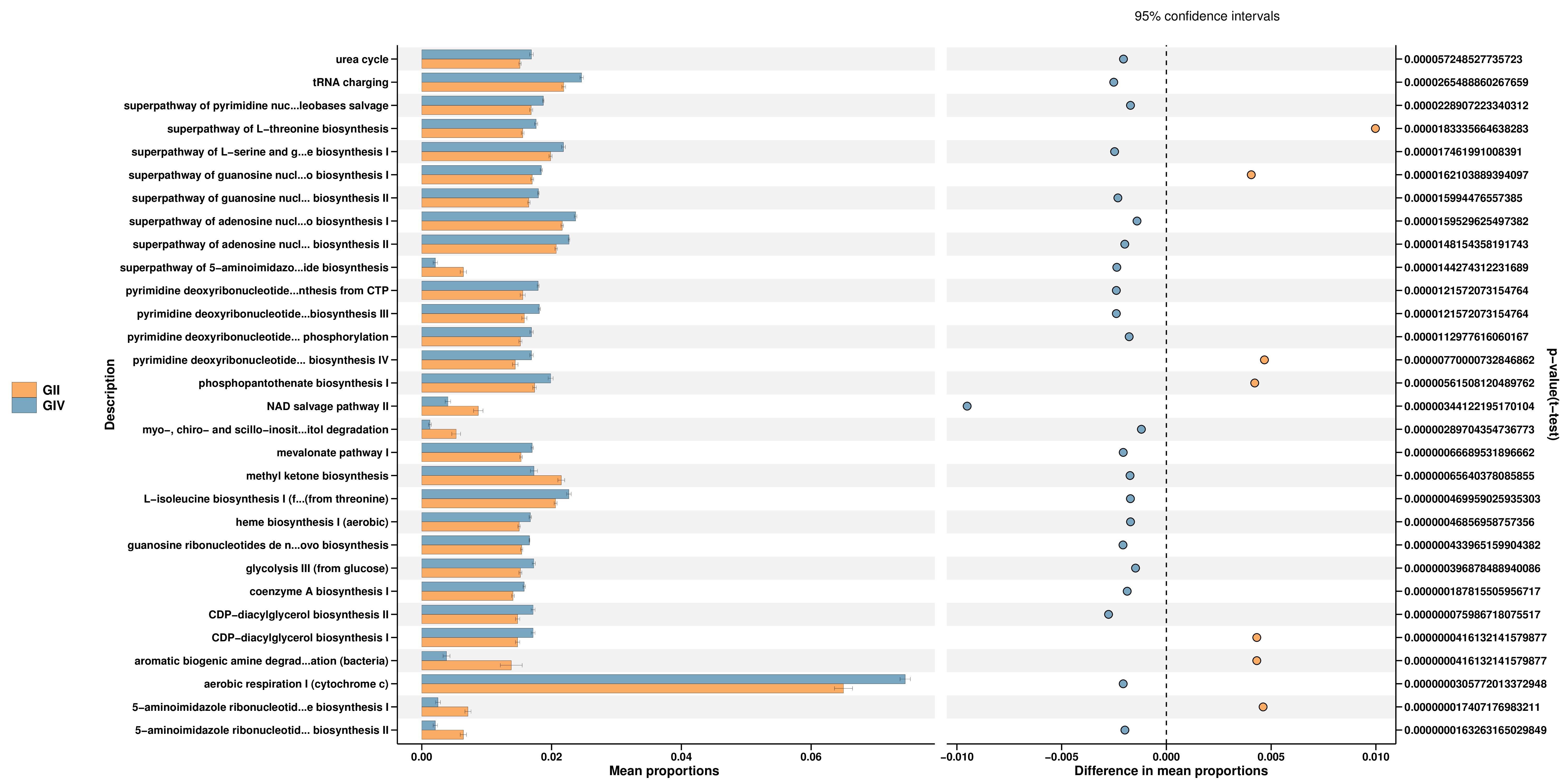

Supplement: Supplementary file 8 [file Image_8.JPEG]
